# Autoimmune/Inflammatory Syndrome Induced by Adjuvants (ASIA) in a 56-Year-Old Patient With Silicone Breast Implants: A Case Report

**DOI:** 10.7759/cureus.108155

**Published:** 2026-05-02

**Authors:** Victor Hugo Garzón Ortega, Jesús C Ceballos Villalva, Fernando Fernández Varela Gómez, Jaime Enrique Flores Avila, Hernando Alberto Vázquez Sánchez, Alec Anceno Olivares, Daniela Téllez-Palacios

**Affiliations:** 1 Department of General Surgery, Christus Muguerza Hospital Alta Especialidad, Monterrey, MEX; 2 Department of Plastic and Reconstructive Surgery, Hospital General Dr. Manuel Gea González, Mexico City, MEX; 3 Department of Plastic and Reconstructive Surgery, Instituto Nacional de Ciencias Médicas y Nutrición Salvador Zubirán, Mexico City, MEX; 4 Department of Urology, University of Southern California, Los Angeles, USA; 5 Department of Plastic and Reconstructive Surgery, Breast Clinic, Hospital General Dr. Manuel Gea González, Mexico City, MEX

**Keywords:** adjuvant-induced autoimmune syndrome (asia), autoimmune/inflammatory syndrome induced by adjuvants (asia), breast implants, capsular contracture, surgical case report

## Abstract

Autoimmune/inflammatory syndrome induced by adjuvants (ASIA) is a clinically complex condition triggered in genetically predisposed individuals by substances such as silicone in breast implants. Although silicone implants have traditionally been considered inert, increasing evidence links them to autoimmune phenomena. This report presents a 56-year-old woman who experienced progressive muscle weakness, hand and facial edema, alopecia, and elevated creatine kinase levels one year after silicone implant exchange. A diagnosis of ASIA, complicated by Grade IV capsular contracture, was established based on clinical, serological, and dermatological findings. Despite initial immunosuppressive therapy, symptoms persisted, prompting explantation and mastopexy. This case underscores the value of a multidisciplinary approach and highlights explantation as a potential strategy for improving ASIA-related outcomes.

## Introduction

Autoimmune/inflammatory syndrome induced by adjuvants (ASIA) encompasses a broad range of immune-mediated manifestations in genetically susceptible individuals following exposure to adjuvants that enhance the immune response [1-3]. These adjuvants may include silicone, metals, or other materials used in cosmetic and reconstructive procedures. Although silicone implants have long been regarded as relatively inert, multiple clinical reports link them to phenomena consistent with ASIA. The main mechanisms of the development involve chronic hyperstimulation of the innate immune system by the adjuvant, leading to loss of self-tolerance through molecular mimicry, epitope spreading, and bystander activation, resulting in autoantibody production, cytokine dysregulation, and progression to overt autoimmune diseases [2,4,5].

Despite limited large-scale epidemiological data, often affected by publication bias, recognition of ASIA has grown [4-6]. In a retrospective Mexican study of 1,027 cases involving the injection of foreign substances for cosmetic purposes, many patients presented with local symptoms, and some developed systemic features suggestive of an adjuvant-driven inflammatory process [7]. While that study did not focus solely on breast implants, it highlights the possibility that silicone and other materials may act as adjuvants in susceptible individuals [1,7].

Patients with implant-associated ASIA frequently present with fatigue, arthralgias, cognitive impairment, sicca manifestations, and autoantibody production (e.g., antinuclear antibodies (ANA)), complicating diagnosis [2,6,8,9]. Symptom onset may range from months to years after implantation, warranting careful clinical assessment [2]. Patients with preexisting autoimmune disorders or a family history of such conditions may face a higher risk [4-6].

Herein, we describe a 56-year-old patient with silicone implants who developed cutaneous, muscular, and systemic signs consistent with ASIA. By correlating elevated creatine kinase (CK) levels, alopecia, and severe capsular contracture, the case illustrates the diagnostic challenges surrounding ASIA and underscores potential therapeutic considerations.

## Case presentation

**Figure 1:**
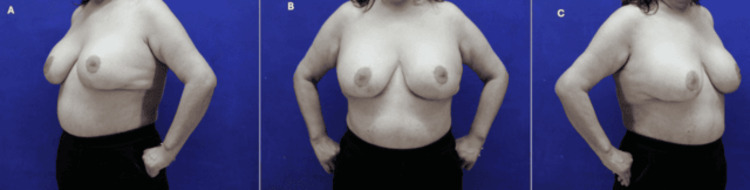
Preoperative images demonstrating Grade IV capsular contracture from multiple angles (A) Left oblique view of the patient’s left breast, showing marked upper pole tightness and obvious distortion consistent with Grade IV capsular contracture. (B) Frontal view, highlighting asymmetry between the two breasts and a significantly firm, contracted appearance on the patient’s left side. (C) Right oblique view, again illustrating the severity of the contracture and the marked displacement of the breast implant

A 56-year-old woman from Mexico City, with hypothyroidism, underwent abdominal liposuction and breast augmentation in 2002. In 2021, she received a silicone implant exchange at our institution (the original implants had a smooth surface, whereas the replacement implants were nanotextured, with differences in gel cohesivity; however, the potential for immune stimulation associated with silicone exposure remained). One year later, she developed progressive muscle weakness, facial and hand edema, and persistent digital hyperemia. Laboratory evaluations revealed elevated CK, positive ANAs (1:1280) with negative specificities, and Grade IV capsular contracture (Figure 1).

**Figure 2:**
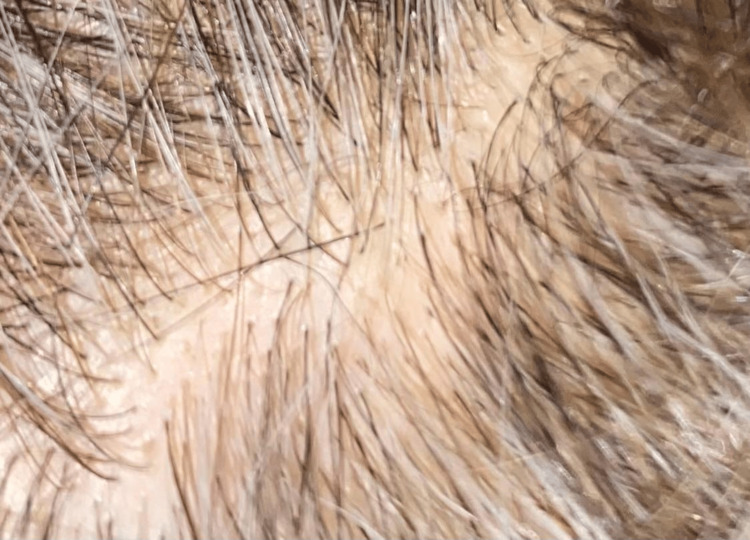
Telogen effluvium on the patient’s scalp This close-up photograph reveals diffuse hair thinning and increased scalp visibility, consistent with telogen effluvium diagnosed during dermatological evaluation. The findings align with the patient’s broader presentation of autoimmune/inflammatory features in ASIA, where inflammatory triggers may contribute to premature hair shedding

A summary of the patient’s laboratory tests is provided in Table 1. Based on clinical and serological findings, a diagnosis of ASIA was considered. She initially received methotrexate and prednisone; however, methotrexate was discontinued due to intolerance, and azathioprine was added alongside a prednisone taper. Dermatological evaluation identified telogen effluvium (Figure 2) and erythematous papules on the dorsal hands, managed with topical corticosteroids and photoprotection.

**Table 1:** Relevant laboratory parameters The table shows the patient’s laboratory parameters on two separate dates (January 17 and February 15, 2023). While the white blood cell count remained within normal limits, there was an initial predominance of neutrophils that shifted toward a higher lymphocyte percentage over time. Notably, CK was elevated (300 U/L) initially but normalized (105 U/L) by the second test, and CRP remained elevated at both points. The patient also had consistently high antinuclear antibody titers (1:1280), underscoring an ongoing inflammatory or autoimmune component consistent with ASIA CK: creatine kinase; CRP: C-reactive protein; ASIA: autoimmune/inflammatory syndrome induced by adjuvants; TPO: thyroid peroxidase

Parameter	Normal range	November 19, 2022	January 17, 2023	February 15, 2023
White blood cells (×10³/µL)	4.0-10.0	-	6.3	5.9
Neutrophils (%)	40-75	-	75.3	62.7
Lymphocytes (%)	20-45	-	19.7	33.3
Monocytes (%)	2-10	-	4.2	2.2
Eosinophils (%)	0-6	-	0.2	1.0
Basophils (%)	0-2	-	0.6	0.8
Hemoglobin (g/dL)	13.5-17.5 (M)/12-16 (F)	-	15.8	14.6
Hematocrit (%)	38-50	-	45.2	43.2
Red cell distribution width (%)	11.5-14.5	-	-	14.8
Platelets (×10³/µL)	150-400	-	215	207
Blood urea nitrogen (mg/dL)	7-20	-	16.1	17.3
Urea (mg/dL)	15-40	-	34	37
Creatinine (mg/dL)	0.6-1.3	-	-	0.47
Uric acid (mg/dL)	3.4-7.0	-	-	5.0
CK (U/L)	30-200	-	300	105
CRP (mg/dL)	<0.5	-	168	189
Folate (ng/mL)	>3.0	-	-	20.88
Vitamin B12 (pg/mL)	200-900	-	-	222
Thyroid-stimulating hormone (uIU/mL)	0.4-4.0	-	-	1.31
Free T4 (ng/dL)	0.8-1.8	-	-	0.84
Thyroglobulin antibodies (IU/mL)	<4.1	-	-	16.5
Anti-TPO antibodies (IU/mL)	<9.0	-	-	1.20

**Figure 3:**
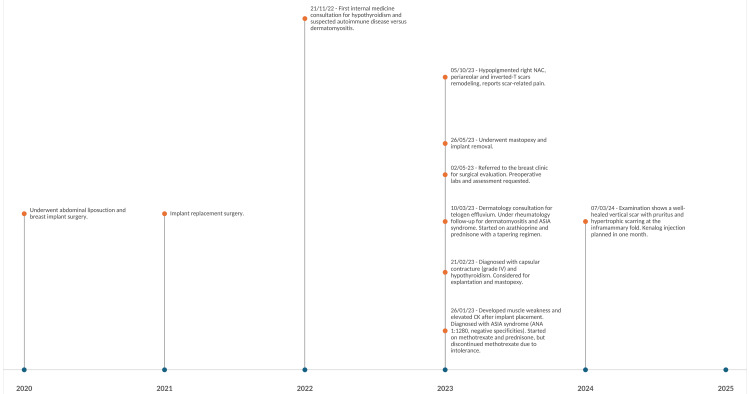
Timeline of clinical events (onset of symptoms, implant exchange, and explantation) ASIA: autoimmune/inflammatory syndrome induced by adjuvants; ANA: antinuclear antibodies; CK: creatine kinase; NAC: nipple areola complex

Because of persistent symptoms and capsular contracture, explantation and mastopexy were performed in May 2023. Postoperatively, muscle weakness partially improved, but pruritus and hypertrophic scarring emerged, prompting consideration of intralesional corticosteroids. The patient remains under follow-up with dermatology and rheumatology. Figure 3 depicts a detailed timeline of clinical events.

## Discussion

We report a patient with a prolonged history of silicone breast implants who manifested progressive muscle weakness, alopecia, dermatological findings, and Grade IV capsular contracture-features collectively suggestive of an ASIA-like inflammatory process [1,2].

Controversies persist regarding the pathophysiology and true incidence of silicone-related immune dysregulation [1,10-16]. While some individuals experience clinical improvement after explantation, outcomes are heterogeneous, possibly reflecting genetic predisposition or coexisting autoimmune disease [12]. Capsular contracture severity, as observed here, may mirror an exaggerated local immune response, paralleling systemic manifestations. The patient’s partial improvement following explantation aligns with observations in other ASIA cases, although symptom resolution varies widely [1,2].

This case highlights a rare but increasingly acknowledged association between silicone implants and ASIA, particularly where severe contracture and dermatological symptoms, such as telogen effluvium, are present. Nevertheless, telogen effluvium in ASIA cases may also arise independently from the chronic inflammatory state, cytokine dysregulation, or other associated factors. These cutaneous manifestations expand the recognized clinical spectrum of ASIA and underscore diagnostic challenges, notably in distinguishing it from dermatomyositis (characterized by hallmark rashes including Gottron’s papules and heliotrope rash) [17,18] or from severe hypothyroid presentations like myxedema coma, which involves distinct metabolic derangements (e.g., bradycardia and hypothermia) [19,20]. Table 2 compares ASIA with these conditions, illustrating why an ASIA diagnosis is most plausible in this scenario.

**Table 2:** Comparative overview of ASIA, DM, and myxedema coma ASIA: autoimmune/inflammatory syndrome induced by adjuvants; EULAR: European League Against Rheumatism; ACR: American College of Rheumatology classification; HLA: human leukocyte antigen; CK: creatine kinase; EMG; electromyography; ANA: antinuclear antibody; TSH: thyroid-stimulating hormone; SLE: systemic lupus erythematosus; DM: dermatomyositis Source: [3,4,8,13-21]

Aspect/criterion	ASIA (Shoenfeld and Agmon-Levin)	DM (2017 ACR/EULAR)	Myxedema coma (severe hypothyroidism)	Patient’s case
1. Etiopathogenic factor/exposure	Exposure to adjuvants (e.g., silicone) - genetic predisposition (e.g., HLA) [3,13,21]	Autoimmune/paraneoplastic etiology - usually not tied to an external agent [14]	Severe hypothyroidism (any cause); no adjuvant link required [15,16]	Silicone implants replaced in 2021; symptoms began ~1 year afterward
2. Specific cutaneous findings	Nonspecific rashes (vasculitis-like), pruritus, telogen effluvium [3,21]	Gottron’s papules, heliotrope rash, “shawl sign” [17,18]	Myxedema changes: thick, dry, cold skin; periorbital edema, possible scaling [19,20]	Telogen effluvium (alopecia), some erythema on hands; no Gottron’s or heliotrope rash; no typical myxedematous changes
3. Muscle weakness/myalgias	Common complaints: generalized muscle aches, fatigue [21]	Symmetric, proximal muscle weakness - elevated CK, EMG/biopsy inflammation [14]	Weakness from hypometabolism; often with hyporeflexia, muscle cramps [20]	Progressive muscle weakness; initial CK of 300 U/L; no EMG or biopsy performed
4. Laboratory findings	ANA often positive; nonspecific inflammatory markers [3,13,21]	Myositis-specific antibodies (e.g., anti-Mi-2) [18]	High TSH, low T4 in severe cases; hyponatremia, hypercapnia, etc. [15,19]	ANA 1:1280, negative specificities; CK normalized to 105 U/L; normal TSH, normal T4L
5. Inclusion criteria	1: Adjuvant exposure, 2: autoimmune symptoms, 3: improvement after adjuvant removal, 4: genetic risk [3,13,21]	EULAR/ACR 2017 criteria	Scoring systems [15,16]	Meets: silicone exposure + inflammatory signs; partial improvement postexplant
6. Exclusion criteria	A clearly defined autoimmune disease (like SLE or confirmed DM) could exclude ASIA [3,13,21]	Lack of hallmark rash or normal EMG/biopsy argues against DM [17,18]	Normal thyroid function or lack of severe hypothyroid signs (coma, hypothermia) rules it out [15,19]	DM: no typical rash or biopsy; myxedema coma: TSH normal, no severe hypothyroid status
7. Key findings/final comment	ASIA fits best; silicone exposure; elevated ANA; muscle/skin complaints	No confirmatory DM rash or antibody pattern [4,8], CK not extremely high, no EMG/biopsy [14,18]	Normal TSH, no hypothermia/confusion [15,16]; lacks classic myxedema coma traits	Diagnosis: ASIA is the most consistent; DM unlikely; myxedema coma ruled out

The diagnosis of ASIA remains controversial due to a lack of definitive biomarkers [10,11]. While Shoenfeld et al. proposed major and minor criteria, quantitative validation is limited [3,13,15]. Further investigation is required to clarify its immunopathological mechanisms [21].

Limitations

Because this is a single case, it cannot establish a definitive causal relationship between silicone implants, capsular contracture, and ASIA. Confounding factors, including the patient’s hypothyroidism and previous surgeries, may also affect interpretation. Prospective studies are warranted to elucidate the interplay among breast implants, ASIA, and comorbid autoimmune conditions [9,12].

Ultimately, heightened awareness of ASIA is essential for patients presenting with systemic or dermatological symptoms following implantation. Explantation may be beneficial in refractory cases, though responses can vary. Future research should explore capsular tissue immunology, identify practical biomarkers, and assess long-term outcomes postexplantation [9,12]. A coordinated, multidisciplinary approach involving plastic surgeons, rheumatologists, and immunologists is vital to refining both diagnostic and therapeutic strategies. We also recognize that delayed presentations have been described and that temporal association alone does not prove causation.

## Conclusions

This case illustrates how Grade IV capsular contracture may represent an immune-mediated manifestation within the broader spectrum of ASIA, triggered in part by silicone breast implants. In addition to muscle weakness and alopecia, the severity of the contracture underscores a possible adjuvant-driven inflammatory process. Although explantation provided partial symptomatic relief, further research is needed to clarify the direct role of capsular contracture in ASIA pathogenesis and to determine long-term clinical outcomes. A multidisciplinary approach remains key to optimizing diagnosis and management.
